# Integrating impulsivity, emotional regulation, and latent behavioural profiles to predict adolescent mental health outcomes: A Comprehensive behavioural analysis

**DOI:** 10.1192/j.eurpsy.2025.660

**Published:** 2025-08-26

**Authors:** M. Grigoriou, J. von Trott, P. Lalousis

**Affiliations:** 1 Department of Psychology, University of Limassol, Limassol, Cyprus; 2 Institute of Psychiatry, Psychology & Neuroscience, King’s College London, London, United Kingdom; 3 Department of Psychiatry and Psychotherapy, Ludwig-Maximilian-University Munich, Munich, Germany

## Abstract

**Introduction:**

Adolescence is a pivotal phase for behavioural development, where impulsivity, risk-taking, and emotional regulation mechanisms differentially impact mental health outcomes. This study examines the interaction of these factors in predicting addiction and behavioural disorders, focusing the identification of hidden behavioural profiles. The results seek to guide specific interventions for adolescents at risk.

**Objectives:**

This study aims to: (1) investigate the predictive influence of impulsivity, risk-taking behaviours, and emotional regulation on addiction and mental health disorders in adolescents; (2) delineate distinct behavioural profiles using clustering analysis; and (3) recommend intervention strategies informed by these behavioural profiles and gender disparities.

**Methods:**

Data were obtained from 853 adolescents aged 12 to 17 years from the NKI Rockland Sample, a continuous, institutionally focused initiative designed to establish a large-scale lifespan sample. Behavioural features were assessed via the UPPS-P Impulsive Behaviour Scale, the Emotional Regulation Questionnaire (ERQ), and the Youth Risk Behaviour Surveillance System. Clustering analysis, namely K-means and hierarchical methods, was employed to discern latent behavioural characteristics. Logistic regression and random forest models forecasted addiction and mental health outcomes, whereas time series analysis investigated emotional regulation trajectories across clusters.

**Results:**

Clustering analysis revealed four distinct behavioural profiles: Cluster 1 (27%) exhibited few behavioural issues, Cluster 2 (15%) showed high levels of impulsivity and emotional dysregulation, Cluster 3 (38%) had moderate behavioural issues, and Cluster 4 (20%) had moderate-to-high behavioural and emotional difficulties. Emotional regulation trajectories indicated that cognitive reappraisal increased with age (mean score of 28 at ages 12-13 vs. 32 at 16-17), while expressive suppression decreased (mean score of 14 at ages 12-13 vs. 10 at 16-17) (Figure 1). Cognitive reappraisal was significantly associated with better behavioural outcomes, including lower hyperactivity (r = -0.45, p < 0.01) and aggression (r = -0.38, p < 0.01), particularly in females.

**Image 1:**

**Figure:**
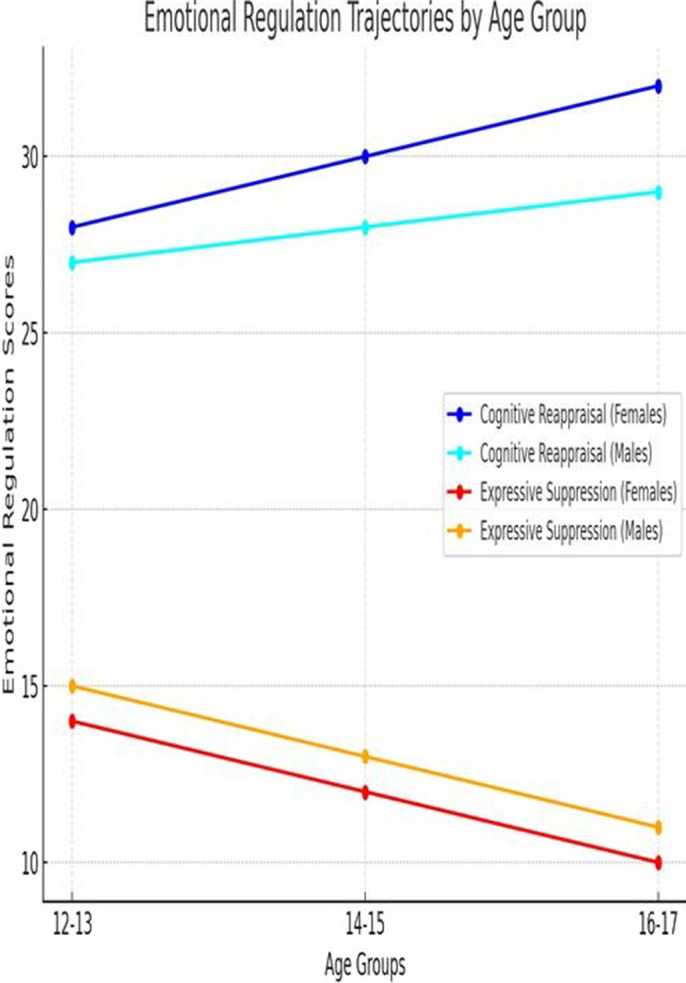

**Conclusions:**

The interplay of impulsive traits, emotional regulation techniques, and risk behaviours is crucial in forecasting mental health consequences in teenagers. The recognition of unique behavioural profiles and gender-specific variations highlights the necessity for individualised interventions. Assessment of high-risk profiles, especially those characterised by elevated impulsivity and emotional dysregulation, along with the encouragement of cognitive reappraisal as a regulatory approach, can substantially reduce the likelihood of behavioural disorders and addiction.

**Disclosure of Interest:**

None Declared

